# Bird Species Identification Using Spectrogram Based on Multi-Channel Fusion of DCNNs

**DOI:** 10.3390/e23111507

**Published:** 2021-11-13

**Authors:** Feiyu Zhang, Luyang Zhang, Hongxiang Chen, Jiangjian Xie

**Affiliations:** School of Technology, Beijing Forestry University, Beijing 100083, China; zfy15810038216@163.com (F.Z.); zly12730172@163.com (L.Z.); millerchx@gmail.com (H.C.)

**Keywords:** bird vocalization, spectrogram feature, multi-channel, deep convolutional neural

## Abstract

Deep convolutional neural networks (DCNNs) have achieved breakthrough performance on bird species identification using a spectrogram of bird vocalization. Aiming at the imbalance of the bird vocalization dataset, a single feature identification model (SFIM) with residual blocks and modified, weighted, cross-entropy function was proposed. To further improve the identification accuracy, two multi-channel fusion methods were built with three SFIMs. One of these fused the outputs of the feature extraction parts of three SFIMs (feature fusion mode), the other fused the outputs of the classifiers of three SFIMs (result fusion mode). The SFIMs were trained with three different kinds of spectrograms, which were calculated through short-time Fourier transform, mel-frequency cepstrum transform and chirplet transform, respectively. To overcome the shortage of the huge number of trainable model parameters, transfer learning was used in the multi-channel models. Using our own vocalization dataset as a sample set, it is found that the result fusion mode model outperforms the other proposed models, the best mean average precision (MAP) reaches 0.914. Choosing three durations of spectrograms, 100 ms, 300 ms and 500 ms for comparison, the results reveal that the 300 ms duration is the best for our own dataset. The duration is suggested to be determined based on the duration distribution of bird syllables. As for the performance with the training dataset of BirdCLEF2019, the highest classification mean average precision (cmAP) reached 0.135, which means the proposed model has certain generalization ability.

## 1. Introduction

Birds have been widely regarded as important indicators of biodiversity [1], thus, it is significant to monitor bird species. At the species level, bird vocalizations are relatively stable, which can be used for species identification [2,3]. Furthermore, it is possible to monitor bird species by using autonomous recording units [4,5]. Considering the large amount of audio data from long-term recording programs, an efficient, automated identification method of bird species should be induced to shorten the analysis time and decrease the workload.

The spectrogram of bird sounds represents the intensity of the sound signal by different colors or gray values, which contains the time–frequency feature of bird sounds. In the spectrogram, bird vocalization can be seen as a kind of special object. Then, we can identify bird species through the image features of the special object. The key point of automated bird species identification is the extraction of identifiable features of bird vocalizations. Deep learning has a strong ability of self-learning and feature extraction, which can automatically acquire characteristic information from inputs [6]. Koops et al. [7] trained eight deep neural networks with the inputs of mel-frequency cepstral coefficients (MFCCs) of bird audio segments. The results showed that the best network classified 73% correctly. Piczak [8] studied three different DCNNs and a simple ensemble model to complete the LifeCLEF 2016 bird identification task. The highest mean average precision (MAP) of 52.9% was achieved for foreground species. Toth and Czeba [9] fed the spectrograms into a convolutional neural network (CNN) to realize the classification of bird species. The solution reached the MAP score of 40% for main species. When there were background species, the MAP score of 33% was achieved. Sprengel et al. [10] processed the background noise by image process methods before feeding into the CNN. The MAP score of 0.686 was achieved when identifying the main species. When background species were considered as additional prediction targets, the MAP score decreased to 0.555. Cakir et al. [11] proposed the convolutional recurrent neural networks to realize automated bird audio detection and achieved an 88.5% Area Under ROC Curve (AUC) score on the unseen evaluation data. Ágnes et al. [12] presented a CNN system to classify bird sounds with a spectrogram as input; when using an RGB spectrogram, the highest accuracy was about 82%. Xie et al. [13] proposed a bird call classification model based on VGG16 with three types of spectrogram; the MAP reached 0.9871 when classifying 18 bird species. Xie et al. [14] utilized an autoencoder improved by self-attention as the acoustic classifier of *Nipponia Nippon* individuals: the highest accuracy was 0.971. Kahl et al. [15] designed the BirdNET with a series of residual stacks and classification blocks, aiming to identify 984 bird species by the Fast Fourier Transform (FFT) spectrograms of bird vocalizations. The MAP of 0.791 was achieved for single-species recordings. Turker et al. [16] introduced the discrete wavelet transform (DWT) to calculate the spectrogram, which was used to classify the bird sound. The accuracy of 96.67% was achieved on the 18 classes bird sound dataset. All of the above studies show that the bird species identification methods based on deep learning are effective. Through designing a reasonable network architecture and selecting appropriate input features, the identification accuracy of deep learning can surpass other classification methods.

In the wild, the sample size of rare bird vocalizations may be limited. Moreover, there are regional differences among the vocalizations of birds in different places. Therefore, we should not simply download their vocalizations from websites as the training data. In this situation, the sample size of bird vocalizations is relatively small compared to the demands of a deep neural network model, which tends to cause the overfitting problem when training the neural network model. Transfer learning extracts features from a pretrained model, which decreases the number of trainable parameters significantly, then reduces the demand for the number of samples [17,18]. Thus, it can avoid overfitting. Atoine [19] proposed an efficient bird sound classification method: Soundception, which was realized by the transfer learning of Inception-V4. Soundception reached the MAP of 0.714 in the task of classifying of 1500 bird species.

In this paper, we proposed a DCNN model to overcome the imbalance of dataset and studied the performance of two multiple model fusion schemes in bird vocalization classification, finding the best fusion strategy. The main contributions of this paper are as follows:

(1) Considering the imbalance of the bird vocalization dataset, a single feature identification model (SFIM) was built with residual blocks and modified, weighted, cross-entropy function. Three SFIMs were trained with three kinds of spectrograms calculated by short-time Fourier transform, mel-frequency cepstrum transform and chirplet transform, respectively.
(2) To achieve better performance, two multi-channel fusion models using three different SFIMs were studied. Furthermore, transfer learning was introduced to decrease the number of trainable parameters of fusion models. The resulting fusion mode model outperforms the feature fusion mode model and SFIMs, the best mean average precision (MAP) reaches 0.914.
(3) Through the comparative experiments with different durations of spectrograms, the results revealed that the duration is suggested to be determined based on the duration distribution of bird syllables.

The rest of this paper is as follows. Section 2 describes the dataset and the proposed bird vocalization identification models. Section 3 presents and discusses the experimental results. Finally, Section 4 gives a brief summary of this paper.

## 2. Materials and Methods

### 2.1. Datasets

#### 2.1.1. Vocalization Signals

In the breeding season, we recorded the vocalization of birds at Beijing Song-Shan National Nature Reserve (east longitude 115°43′44″–115°50′22″, north latitude 40°29′9″–40°33′35″) with digital solid-state recorder Marantz PMD-671 (MARANTZ, Japan) and directional microphone Sennheiser MKH416-P48 (SENNHEISER ELECTRONIC, German) for many years. The vocalization signals are in 16-bit linear WAV format with 44.1 kHz sampling rate. In this paper, we selected the vocalization signals of eighteen bird species, which have been clearly identified by ornithologists. Each signal only contains the vocalization of one species, and there is no overlap between vocalizations. Table 1 lists the detailed information of eighteen bird species. The column of time means the cumulative time of the vocalization signals.

#### 2.1.2. Signal Pre-Processing

Bird vocalization signal is a kind of non-stationary signal. Before the time–frequency transform, pre-processing is needed. Pre-emphasis filter is used to compensate for the high frequency attenuation of vocalization signal at first. The pre-emphasis coefficient was set to 0.95. After that, the vocalization signal was segmented into frames and windowed using the Hamming window function. We chose the frame length of 50 ms to make sure that at least one fundamental frequency peak was included, and 30% overlap was chosen to divide the vocalization signal into windowed frames. The primary element of bird vocalization is ‘notes’ that can be combined into syllables, which, in turn, constitute song types. Acoustic classification of bird species mainly focuses on the classification of individual syllables [20]. Segmenting vocalization into distinct syllables is a crucial step. We performed the segmentation operation in the time domain based on energy, the frames with high energy (higher than a half of the maximum energy) are considered to be syllables, otherwise there is silence.

#### 2.1.3. Spectrogram Calculation

Acoustic signals are usually transformed to spectrograms, which can be used to characterize the time–frequency characteristics of bird vocalization. Bird vocalization can be regarded as the special object in spectrogram, where the characteristic of special object represents the time–frequency characteristic of the bird vocalization.

Here, three most frequently used time–frequency transform methods in the audio signal processing, short-time Fourier transform (STFT), mel-frequency cepstral transform (MFCT) and chirplet transform (CT) were utilized to calculate the spectrograms. STFT is one of the earliest time–frequency analysis methods, which presents the energy distribution across linear range of the frequencies. MFCT was proposed to approximately represent the logarithmic frequency sensitivity of human hearing. We calculated 32-dimensional MFCCs through MFCT, the last 31 dimensions were composed to form the Mel spectrograms. CT is a kind of linear time–frequency representation, which refers to the time–frequency representations of each atom on the modulated time–frequency plane. It is a broad class of filters, which include wavelets and Fourier bases as particular cases, and there is an obvious advantage in the representation of short-time stationary signal [21]. We carried out the CT on each frame with fast chirplet decomposition algorithm [22], then, the calculated wavelet coefficients were used to compose the chirplet spectrogram.

#### 2.1.4. Create Sample Sets

**Figure 1:**
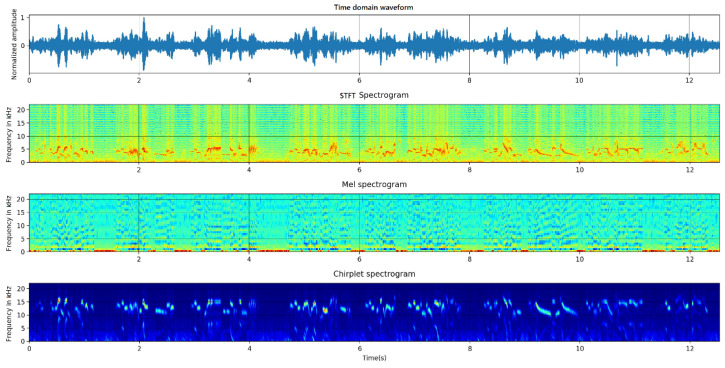
Spectrogram of *Phoenicurus auroreus* (from up to down, they are time domain waveform, STFT spectrogram, mel spectrogram and chirplet spectrogram.).

We utilized the above three kinds of time–frequency transforms to calculate the spectrograms. Figure 1 represents the signal and its spectrograms of *Phoenicurus auroreus*.

Bird species identification is always regarded as the classification of individual syllable types [20]. Hence, the spectrograms of a certain duration were saved as the 224 × 224 RGB color images, instead of the spectrograms of the whole bird vocalization signal. These spectrograms formed the sample set, which will be fed into the identification model. Later, we will discuss the influence of different durations on the performance of the identification models. With three kinds of time–frequency transform methods, three different sample sets with the same size can be built.

### 2.2. Identification Models

#### 2.2.1. Single Feature Identification Model (SFIM) Based on DCNN

**Figure 2:**
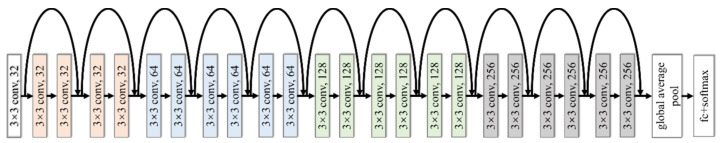
Framework of the single feature identification model.

With the spectrogram of bird vocalization as the input, bird species identification can be thought as an image classification problem. DCNNs can self-learn the image features via some convolutional and pooling layers, then classify the features by some fully connected layers to realize the classification of images [23,24]. The deep residual network (ResNet) is a kind of DCNN model, which yields high performance in the imagenet large scale visual recognition challenge (ILSVRC) of 2015, the top-5 error rate of which was 3.57% [25]. This has been widely used in the field of image recognition [26,27,28]. Compared with the common DCNN, the main innovation of ResNet is the identity shortcut connection, which was adopted to address the degeneration problem of deeper networks. The simple modification can greatly increase the training speed of the model and improve the training performance without raising extra parameters. We constructed single feature identification model with several residual blocks, its framework is shown in Figure 2, and its configuration is shown in Table 2. N is the number of bird species to be classified.

$\left[\begin{array}{l} \mathrm{conv},3\times 3,32,\mathrm{stride}=1\\ \mathrm{conv},3\times 3,32,\mathrm{stride}=1 \end{array}\right]\times 2$ $\left[\begin{array}{l} \mathrm{conv},3\times 3,64,\mathrm{stride}=2\\ \mathrm{conv},3\times 3,64,\mathrm{stride}=1 \end{array}\right]$ $\left[\begin{array}{l} \mathrm{conv},3\times 3,64,\mathrm{stride}=1\\ \mathrm{conv},3\times 3,64,\mathrm{stride}=1 \end{array}\right]\times 2$ $\left[\begin{array}{l} \mathrm{conv},3\times 3,128,\mathrm{stride}=2\\ \mathrm{conv},3\times 3,128,\mathrm{stride}=1 \end{array}\right]$ $\left[\begin{array}{l} \mathrm{conv},3\times 3,128,\mathrm{stride}=1\\ \mathrm{conv},3\times 3,128,\mathrm{stride}=1 \end{array}\right]\times 2$ $\left[\begin{array}{l} \mathrm{conv},3\times 3,256,\mathrm{stride}=2\\ \mathrm{conv},3\times 3,256,\mathrm{stride}=1 \end{array}\right]$ $\left[\begin{array}{l} \mathrm{conv},3\times 3,256,\mathrm{stride}=1\\ \mathrm{conv},3\times 3,256,\mathrm{stride}=1 \end{array}\right]\times 2$

#### 2.2.2. Multi-Channel Identification Models

**Figure 3:**
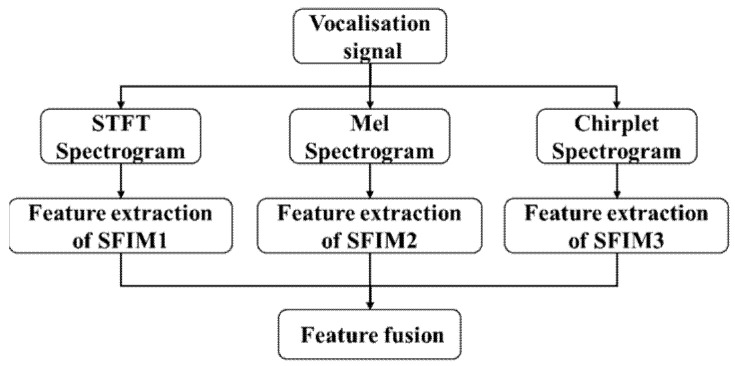
The structure of feature fusion model.

With three kinds of spectrograms as inputs, three different single feature identification models (SFIMs) can be achieved. We further fused three SFIMs together to improve the efficiency and accuracy of the identification. Here, each SFIM is separated to two parts: the feature extraction part and the classifier part. The classifier part only contains the full connect layers and softmax layer. Two fusion modes were designed, one is feature fusion mode, which fuses directly the feature outputs of three SFIMs, its structure is shown in Figure 3.

**Figure 4:**
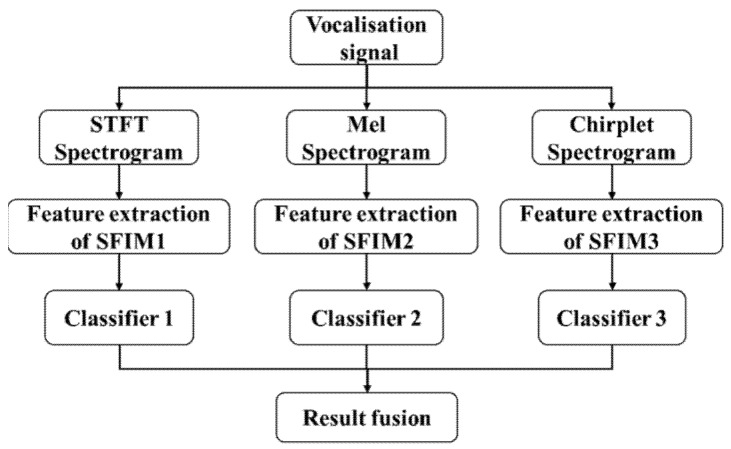
The structure of multi-channel identification models.

The other is result fusion mode, which fuses the classifier outputs of three SFIMs, its structure is shown in Figure 4.

Adaptive linear weighted method is utilized to fulfil the fusion operation, which can ensure that the dimensions of 1 output and input are the same without adding model parameters. The fusion operations of both fusion modes are the same. The fused feature *F* is given by

(1) $$F=\sum_{n=1}^{3}\omega^{n}y^{n}$$

where $\omega^{n}$ and $y^{n}$ are the weight and the vector to be fused of feature *n*. Different weights indicate the contribution of different vectors in the identification, and $\sum_{n=1}^{3}\omega^{n}=1$ should be satisfied. The weights are updated in the training until the optimal values are achieved.

After the fusion operation, two fully-connected layers and a softmax layer are selected as the classification part to realize the classification. To decrease the number of trainable parameters of fusion models, the parameter-based transfer learning is used here. The parameters of each SFIM were frozen, only the parameters of fusion and classification part were trained.

## 3. Results and Discussion

### 3.1. Experimental Setup

The experiments were conducted on an Ubuntu16.04 Linux workstation with 32 G memory, one E5-2620CPU (6 × 2.1 GHz) and two GTX1080ti GPUs (11 GB memory). The models were programmed based on the deep learning framework Tensorflow1.9.

Because the number of spectrograms in each sample set is not large, only a training and test set were built. Each sample set was randomly split into a training and test set with a ratio of 7:3. Based on these samples, the identification models were trained and verified. The training set was divided into several batches to speed up the training process. The detailed training settings are listed in Table 3. Early stopping was used to avoid overfitting.

As Table 1 shows, the cumulative time of different bird vocalization signals are different; also, the number of spectrograms of different bird species in the sample set is quite different. In other words, sample sets are unbalanced, which is not beneficial to the training of DCNN models [30]. We proposed a kind of weighted cross-entropy as the loss function. The loss function can increase the weights of the bird species that have few samples, so that the problem of unbalanced data can be solved. For multi-class classification, the improved cross-entropy loss of the *i*th class is

(2) $${\mathrm{WCE}}_{i}=-\eta_{i}y_{i}\log{\overset{⌢}{y}}_{i}-(1-y_{i})\log(1-{\overset{⌢}{y}}_{i})$$

where $y_{i}$ represents whether the sample belongs to the *i*th class, its value is 1 when the sample belongs to the *i*th class, otherwise it is 0. ${\overset{⌢}{y}}_{i}$ denotes the prediction probability that the sample belongs to the *i*th class. $\eta_{i}$ is the weight of the *i*th class, which is determined by the following equation

(3) $$\eta_{i}=\frac{1-\beta_{i}}{\beta_{i}}$$

where *β_i_* indicates the ratio of the sample size of the *i*th class to the whole sample size.

The improved cost function is presented as follows

(4) $$\mathrm{Cos}t=\frac{1}{N_{B}}\sum_{j=1}^{N_{B}}\beta_{ij}\cdot {\mathrm{WCE}}_{ij}$$

where *N_B_* is the value of the batch size, the subscript *j* is the index of samples in a batch, the subscript *i* denotes the corresponding value of the sample belonging to the *i*th class. *i* can be any class index of the sample in the current batch.

### 3.2. Different Models with the Same Duration of Spectrogram

In the experiments, there are five models, including three SFIMs, the multi-channel model with result fusion (Re-fuse) and the multi-channel model with feature fusion (Fe-fuse). The spectrogram sample sets of 300 ms duration were used to train all the models. Firstly, three spectrogram sample sets, including Ch, Mel and Spe, were fed to three SFIMs, respectively. After that, we froze the parameters of three SFIMs and trained the Re-fuse and Fe-fuse models with the Ch, Mel and Spe spectrograms at the same time.

Mean average precision (MAP) is commonly used to evaluate the performance of an identification model, which is defined as

(5) $$\mathrm{MAP}=\frac{\sum_{q=1}^{N}AveP(q)}{N}$$

where *q* is the class index and *Ave*P(*q*) is the average identification accuracy of the *q*th class samples.

**Figure 5:**
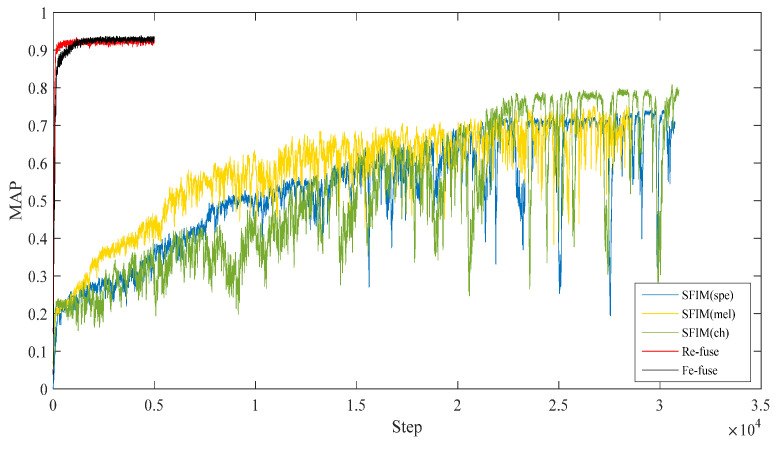
Test MAPs of the proposed models.

Table 4 shows the test MAPs of all the above models. Figure 5 shows the test MAP variations at different steps.

**Figure 6:**
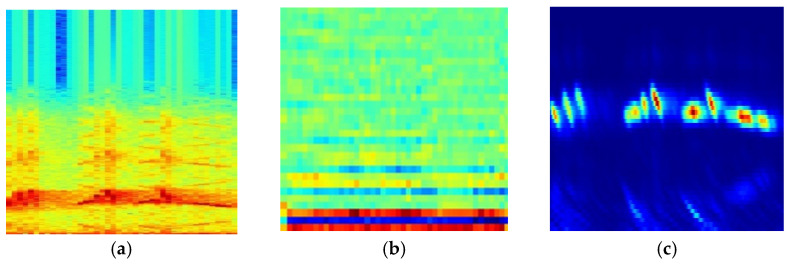
Three kinds of exemplar spectrograms of *Ficedula zanthopygia*. (**a**) Spe spectrogram, (**b**) Mel spectrogram, (**c**) Ch spectrogram.

As shown in Table 4 and Figure 5, it was found that the SFIM(Ch) achieved the highest MAP of 0.808 in three SFIMs, the second was SFIM(Mel), which is consistent with the results of [22]. This demonstrates that the Ch spectrogram is more suitable for bird species identification when using the image classification method. From three kinds of exemplar spectrograms of *Ficedula zanthopygia* listed in Figure 6, it was found that the differences between the bird vocalization region (the redder region) and the background are most obvious in the Ch spectrogram than the other two kinds of spectrograms, and the bird vocalization region of the Ch spectrogram is more compact. All of the above factors enable the best feature extraction ability using the Ch spectrogram as inputs.

As for both multi-channel models, the highest MAPs were higher than those of all the SFIMs. The largest MAP difference is between the Re-fuse model and SFIM (Spe), the MAP of the Re-fuse model is 23.2% higher than SFIM (Spe). The smallest MAP difference is between the Fe-fuse model and SFIM (Ch), and the MAP of the Fe-fuse model is 12.4% higher than SFIM (Ch). The above analysis indicates that using selected multi-features as the input of the network can improve the bird identification accuracy to some extent. Furthermore, because transfer learning was used in the multi-channel models, this shows that the convergences of multi-channel models are much faster than SFIMs, which means that multi-channel models are more efficient. Considering the different fusion modes, the highest MAP of the Re-fuse model is a little higher than that of the Fe-fuse model, and the Re-fuse model arrived at the highest MAP faster. On the other hand, the Re-fuse model has fewer trainable parameters than all of the other models, which is advantageous to realize bird identification when limited samples are available.

### 3.3. Different Models with Different Durations of Spectrogram

**Figure 7:**
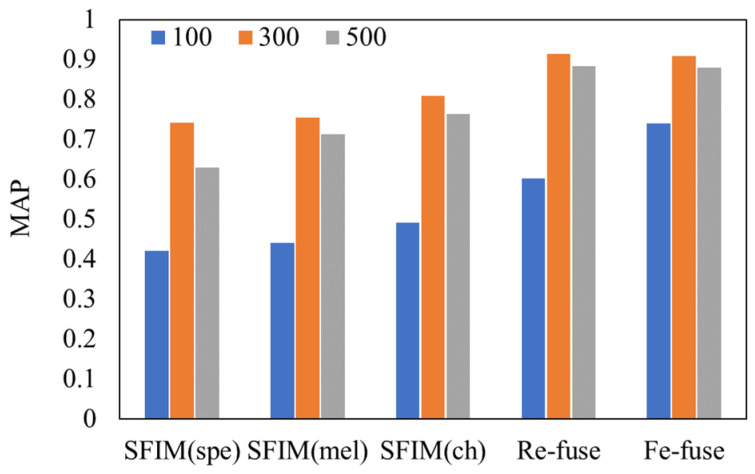
Comparison of test MAP between different models and different durations.

Different durations of spectrogram may affect the performances of the bird identification models. We chose the durations of 100 ms, 300 ms and 500 ms to train the proposed models. A comparison of test MAPs between different models is shown in Figure 7. We found that when the spectrogram sample sets of 300 ms duration were fed in each of the models, the test MAPs were the highest. The worst performances came from the duration of 100 ms.

We concluded the syllable durations of the bird species in our dataset. According to the statistics, the syllable durations of the eighteen bird species are between 100 ms and 250 ms. When 100 ms was chosen as the duration, a part of the syllable may be cut off into different spectrograms so that the complete feature cannot be obtained by the classification of spectrograms. As for 500 ms, the number of samples decreased. Hence, compared with the duration of 300 ms, the identification performances deteriorate with the durations of 100 ms and 500 ms, and the impact in the case of the 100 ms duration is more serious. We suggest that the appropriate duration should be selected according to the duration distribution of the identified bird species.

### 3.4. Performance with BirdCLEF2019 Dataset

The BirdCLEF2019 dataset contains about 350 h of soundscapes, which was built for the 2019BirdCLEF challenge [31]. The labels of the test data were not published. We only used the training data to evaluate our fusion model. The training data covered 659 species from South and North America. Because all data were recorded in the wild, compared with our own dataset and the ICML4 B dataset, there were multi-species, high-level ambient noises in the soundscapes. Due to the above differences, the classification mean average precision (cmAP) was proposed as the evaluation metric in the challenge, considering each class *c* of the ground truth as a query [31]. The cmAP is defined as

(6) $$\mathrm{cmAP}=\frac{\sum_{c=1}^{C}AveP(c)}{C}$$

where *C* is the number of species in the ground truth and *Ave*P(*c*) is the average precision for a given species *c* computed as

(7) $$AveP(c)=\frac{\sum_{k=1}^{n_{c}}P(k)\times \mathrm{rel}(k)}{n_{rel}(c)}$$

where *k* is the rank of an item in the list of the predicted segments containing *c*, *n_c_* is the total number of predicted segments containing *c*, *P*(*k*) is the precision at cut-off *k* in the list, rel*(k)* is an indicator function equaling 1 if the segment at rank *k* is a relevant one and *n_rel_(c)* is the total number of relevant segments for class *c*. The training dataset was randomly divided into ten parts, seven parts were used as training samples, three parts were set as validation samples. During the training step, the duration of 500 ms was selected to compute the spectrograms. The Re-fuse model was trained with the training samples and validated with validation samples.

We performed the experiments five times, the achieved highest cmAP is 0.135, which is a little lower than the results of the ASAS team. The cmAPs of the ASAS team were between 0.140 and 0.160, which made them win the second place [31], and our cmAP is much higher than that of the third place, which is 0.054. The ASAS team used Inception and ResNet architectures and data augmentation to conduct their experiments. They attributed their good result to the sophisticated augmentation strategies. Although the cmAP is a little lower than that of the ASAS team, our model is simpler, the number of trainable parameters is smaller, and we do not need complicated augmentation, which makes our model more efficient. The performance of the Re-fuse model on the BirdCLEF2019 dataset shows that although there are high-level noises, the proposed Re-fuse model also can achieve a relatively high identification accuracy.

## 4. Conclusions

In this study, we proposed the SFIM based on the residual block, then trained three SFIMs with three kinds of spectrograms, Spe, Mel and Ch spectrogram, respectively. Furthermore, based on these three SFIMs, we built two available multi-channel fusion models to improve the identification accuracy of the bird species. Transfer learning was utilized to reduce the size demand of samples. The experiments reveal that the performance of the Re-fuse model is the best compared with other proposed models, the MAP is highest, and the trainable parameter number is the smallest, which leads to the smallest demand of samples. With different durations of spectrograms as inputs, the performances are various. We recommend that researchers choose suitable durations based on the duration distribution features of bird vocalizations to be identified. Our proposed fusion method achieves relatively high performance in the BirdCLEF2019 dataset, and we are also aware that the best cmAP of BirdCLEF2019 is 0.356, which is much higher than ours. Thus, we will try to add other strategies to the Re-fuse model to improve the bird identification performance in the future.

## Figures and Tables

**Table 1:** Vocalization signal details of 18 kinds of bird.

Order	Family	Species	Time (s)
Galliformes	Phasianidae	*Phasianus colchicus*	12
Cuculiformes	Cuculidae	*C. micropterus*	13
		*C. saturatus*	52
		*Cuculus sparverioides*	34
Passeriformes	Corvidae	*Corvus macrorhynchos*	27
		*Urocissa erythroryncha*	96
	Turdidae	*Phoenicurus auroreus*	37
	Muscicapidae	*Ficedula zanthopygia*	61
		*F. narcissina*	82
		*F. elisae*	49
	Paridae	*P. major*	54
		*Parus palustris*	33
		*P. montanus*	38
		*P. venustulus*	26
	Sittidae	*S. villosa*	29
		*Sitta europaea*	36
	Emberizidae	*Emberiza godlewskii*	23
		*E. elegans*	71

**Table 2:** Configuration of the single feature identification model.

Output	Identification Model
224 × 224 × 32	conv, 3 × 3, 32, stride 1
224 × 224 × 32	$\left[\begin{array}{l} \mathrm{conv},3\times 3,32,\mathrm{stride}=1\\ \mathrm{conv},3\times 3,32,\mathrm{stride}=1 \end{array}\right]\times 2$
112 × 112 × 64	$\left[\begin{array}{l} \mathrm{conv},3\times 3,64,\mathrm{stride}=2\\ \mathrm{conv},3\times 3,64,\mathrm{stride}=1 \end{array}\right]$
112 × 112 × 64	$\left[\begin{array}{l} \mathrm{conv},3\times 3,64,\mathrm{stride}=1\\ \mathrm{conv},3\times 3,64,\mathrm{stride}=1 \end{array}\right]\times 2$
56 × 56 × 128	$\left[\begin{array}{l} \mathrm{conv},3\times 3,128,\mathrm{stride}=2\\ \mathrm{conv},3\times 3,128,\mathrm{stride}=1 \end{array}\right]$
56 × 56 × 128	$\left[\begin{array}{l} \mathrm{conv},3\times 3,128,\mathrm{stride}=1\\ \mathrm{conv},3\times 3,128,\mathrm{stride}=1 \end{array}\right]\times 2$
28 × 28 × 256	$\left[\begin{array}{l} \mathrm{conv},3\times 3,256,\mathrm{stride}=2\\ \mathrm{conv},3\times 3,256,\mathrm{stride}=1 \end{array}\right]$
28 × 28 × 256	$\left[\begin{array}{l} \mathrm{conv},3\times 3,256,\mathrm{stride}=1\\ \mathrm{conv},3\times 3,256,\mathrm{stride}=1 \end{array}\right]\times 2$
1 × *N*	global average pool, full connect (fc), softmax

**Table 3:** Training settings.

Items	Value or Method
Batch size	50
Parameter initialization	Random initialization
Optimization algorithm	Adam [29]
Learning rate	0.001
Epochs	100

**Table 4:** MAPs of all the identification models.

Model	MAP
SFIM (Spe)	0.742
SFIM (Mel)	0.754
SFIM (Ch)	0.808
Re-fuse	0.914
Fe-fuse	0.908

## Data Availability

The raw data required to reproduce these findings cannot be shared at this time as the data also forms part of an ongoing study.
